# C-type Lectin Receptors for Tumor Eradication: Future Directions

**DOI:** 10.3390/cancers3033169

**Published:** 2011-08-08

**Authors:** Ingeborg Streng-Ouwehand, Wendy W. J. Unger, Yvette van Kooyk

**Affiliations:** Department of Molecular Cell Biology and Immunology, VU University Medical Center, P.O. Box 7057, 1007 MB Amsterdam, The Netherlands; E-Mails: i.ouwehand@vumc.nl (I.O.-S.), w.unger@vumc.nl (W.W.J.U.)

**Keywords:** dendritic cells, glycans, antibodies, DC-targeting, immunotherapy

## Abstract

Dendritic cells are key regulators in directing immune responses and therefore are under extensive research for the induction of anti-tumor responses. DCs express a large array of receptors by which they scan their surroundings for recognition and uptake of pathogens. One of the receptor-families is the C-type lectins (CLR), which bind carbohydrate structures and internalize antigens upon recognition. Intracellular routing of antigen through CLR enhances loading and presentation of antigen through MHC class I and II, inducing antigen-specific CD4^+^ and CD8^+^ T-cell proliferation and skewing T-helper cells. These characteristics make CLRs very interesting targets for DC-based immunotherapy. Profound research has been done on targeting specific tumor antigens to CLR using either antibodies or the natural ligands such as glycan structures. In this review we will focus on the current data showing the potency of CLR-targeting and discuss improvements that can be achieved to enhance anti-tumor activity in the near future.

## 1. Introduction

The tumor-environment consists of an immune suppressive milieu, leading to tolerance facilitating tumor outgrowth [1]. Many studies focus on reverting this tumor-tolerance into potent cytotoxic immunity mediated by tumor-specific CD8^+^ T-cell and T-helper responses. Furthermore, the induction of efficient memory is needed to eliminate recurrences of the same tumor. Studies are mainly focused on two strategies. One is a passive strategy using the adoptive transfer of tumor-specific CD8^+^ T-cells or tumor associated antigen (TAA)-specific antibodies [2,3]. The second strategy includes active immunisation, where antigen presenting cells (APCs) are loaded with TAA to induce full-blown anti-tumor responses that consist of effective CD4^+^ and CD8^+^ T-cell responses and prolonged memory [4-6]. Both antigen specific CD4^+^ and CD8 T^+^ cells are required to induce an optimal immune response that instructs macrophages, B cells for antibody responses by CD4^+^ T-cells and killing of the tumor cells by CD8^+^ cytotoxic T-cells. Various formulations of antigen are studied for improving antigen presentation by DCs such as full-length protein or long peptides encompassing the immunodominant epitope, which need to be processed, short peptides or RNA encoding for TAA.

Dendritic Cells (DCs) are the most potent APC and key regulators of T-cell responses [7], thereby controlling immune responses against cancer. They take up antigen using a wide array of receptors by which they are able to discriminate between self versus non-self and/or danger versus non-danger. Depending on the presence or lack of these ‘danger’-signals, either immunity or tolerance is induced. Because of these immune-modulatory characteristics DCs have great potential in vaccine development, not only in long-lasting immunogenic responses against pathogens or tumor-cells but also by the induction of tolerance in auto-immunity and allergy. Here, we will concentrate on the use of DCs in anti-tumor vaccine development.

To date, most approaches that are considered as DC-based immunotherapy are concentrated on the transfer of *in vitro* generated antigen-loaded DCs [4-6]. This includes the isolation of monocytes of patients, to develop *in vitro* immature DCs that can be loaded with the desired antigen in MHC class I or II molecules. These immature DC can mature through the addition of a maturation stimulus or danger stimulus, such as certain cytokine cocktails or pathogenic structures, or adjuvants. This maturation creates the optimal DC that expresses many co-stimulatory molecules that are essential for priming and activation of antigen specific T-cells. These antigen-loaded mature DC are given back to the patient for stimulating a tumor antigen specific immune response that ideally stimulates both antigen specific CD4^+^ and CD8^+^ T-cells. Clinical studies performed using this approach showed that although specific immune responses are monitored, patients did not always show a clinical response [8]. The discrepancy between induced immunological responses and poor clinical outcome is not known, although some suggestions have been made. The use of monocyte-derived DCs might not resemble DCs present *in vivo*. These monocyte-derived DCs might not be the right DC initiating CD8^+^ T-cell responses and may have a poor migratory capacity in particular to migrate to the draining lymph node to stimulate antigen specific T-cells. Moreover, *in vitro* generation of antigen-loaded DC is elaborate and it lacks the possibility for mass production techniques. This will pose significant drawbacks for the commercial development of this therapy, decreasing the probability that this technique will be performed at large scale.

A more direct and less laborious technique is to target antigen to DCs *in vivo* via DC-specific receptors. Antigens can be incorporated into antigen delivery systems, such as liposomes or nanoparticles, which is subject of considerable investigation recently.

In this review we discuss the current progress that has been made on the development of *in vivo* DC-targeting strategies.

## 2. C-type Lectin Receptors (CLRs) as Targeting-Receptors

The ideal antigen-targeting receptor for DC should be DC-specific, and not only serve as an efficient uptake vehicle but also modulate the induced immune response towards anti-tumor immunity by inducing CTLs, Th1 responses and secretion of pro-inflammatory mediators. Different receptors are under extensive research with special interest to C-type lectin receptors (CLRs). CLRs are known to recognize carbohydrate structures through single or multiple carbohydrate recognition domains (CRD) [9]. Depending on the structure and gene locations they have been classified into several groups, of which group II, V and VI are highly expressed on antigen presenting cells [10]. Many of those CLRs, endocytose antigens, upon binding of natural ligands or specific antibodies, followed by presentation of antigen to CD4^+^ T-cells. Some of these CLR, like DEC-205, CLEC9A, Langerin and DC-SIGN, are known to skew the internalized exogenous antigen into the cross-presentation route leading to presentation of antigen to CD8^+^ T-cells [11-15]. Furthermore, different CLRs like Dectin-1, CLEC9A and DC-SIGN have signalling-capacities and are able to modulate immune responses upon recognition of endogenous ligands expressed on self- or pathogenic antigens [16,17]. For example, DC-SIGN-mycobacterial ManLAM interaction promotes the production of pro-inflammatory cytokines IL-6 and IL-12 via Raf-1 mediated signalling pathway [18]. In contrast, binding of fucosylated pathogens to DC-SIGN initiates a Raf-1 independent signalling pathway, resulting in strong IL-10 production, and decreased production of IL-6 and IL-12 [19]. Because of their endocytic, cross-presenting and immunomodulatory character these receptors are very interesting to explore for DC-based immmunotherapies. A summary of CLRs used for DC-targeting strategies, with their expression-patterns and function in humans and mice, is highlighted in Table 1.

## 3. Targeting Antigen-Antibody Conjugates to CLR

**Figure 1.**
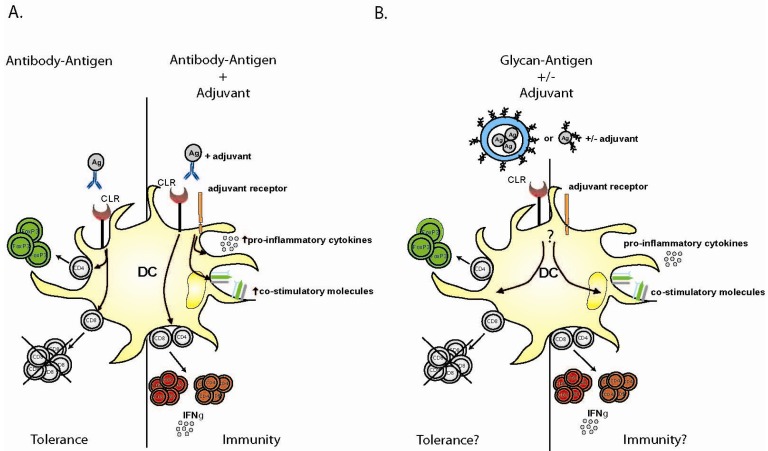
Shaping immune responses using DC targeting antibody-antigen or glycan-antigen conjugates. (A) Targeting of antibody-antigen conjugates to DCs with an additional adjuvant induces upregulation of co-stimulatory molecules, antigen-specific CD8^+^ and/or CD4^+^ T-cell responses and the secretion of pro-inflammatory cytokines. Which response is mediated depends on the DC-subset which expresses the CLR and on the adjuvant that is used. Targeting of antibody-antigen conjugates without an additional maturation stimulus leads to transient proliferation and deletion T-cells and the induction of T-regulatory cells, leading to tolerance; (B) The use of glycan-antigen conjugates can lead to the induction of CD4^+^ and CD8^+^ T-cell responses, depends on the CLR targeted. To date, no T-regulatory cell-induction has been observed when targeting DCs with glycan-antigen conjugates without adjuvant, but this cannot be ruled out for all CLR/glycan combinations. Currently it is unsufficiently known which adjuvant can be optimally combined with the modified antigens to induce the desired immune response. This is currently being investigated.

The first and so far the most extensive studied CLR for antigen-targeting is DEC-205. The targeting of DEC-205 using antibody-antigen conjugates and an additional DC-maturation stimulus (anti-CD40) dramatically enhanced T-cell proliferation, resulting in improved anti-tumor-immunity [11]. The lack of a maturation-stimulus led to initial T-cell division but subsequently T-cell deletion and unresponsiveness in both CD4^+^ and CD8^+^ T-cell compartment was established [20] (Figure 1A). These findings could be confirmed in a mouse model for auto-immune diabetes where both onset and progression of the disease could be inhibited. The targeting of β-cell mimotopes to DEC-205 led to the deletion of auto-reactive CD8^+^ T-cells [52].

One of the advantages for targeting antigen to DEC-205 is that the receptor is not down-modulated upon maturation, making it possible for mature DCs to still take up and present antigens [53]. This could potentially enhance strength and prolong durability of responses, leading to powerful vaccine development. However, most studies to date have been performed in mice and the expression of DEC-205 in humans is more widespread than in mice, since expression of DEC-205 is also found on B-cells, T-cells, monocytes, macrophages and NK cells [54]. It has been shown that *in vitro* targeting antigen- anti-DEC205 single chain fragments variable (scFv) to monocyte-derived DCs derived from melanoma patients leads to efficient CD4^+^ T-cell proliferation [55]. However, whether DEC-205 targeting in humans leads to efficient CD8^+^ T-cell responses *in vivo* remains to be elucidated. The first clinical studies are currently being conducted and will elucidate whether DEC-205 is as potent in humans as in mice.

Next to DEC-205 several other CLRs are being explored for DC-based vaccination, one of which is the recently discovered CLEC9A (also called DNGR-1). CLEC9A is predominantly expressed on murine CD8α^+^ DCs and human BDCA-3^+^ cells [12,15,56], and thus on another subset of DCs than DEC-205. Its physiological function is to take up dead cells for presentation in MHC class I (cross presentation) to stimulate CD8^+^ T-cells [17]; however, the exact molecular ligand on dead cells that is recognized by CLEC9A has not been identified yet. The targeting of antigen to CLEC9A using antibody-antigen conjugates has led to the efficient endocytosis of antigen resulting in enhanced CD4^+^ and CD8^+^ T-cell proliferation and strikingly high antibody responses. Furthermore, the targeting of CLEC9A in mice has shown to be efficacious in tumor rejection [12,15]. Antigen-targeting to CLEC9A without the addition of any maturation-stimulus has been shown to induce CD8α^+^ DC dependent MHC-class II antigen-presentation and drives differentiation of CD4^+^ T-cells to FoxP3^+^ T-regulatory cells in steady-state condition [57] (Figure 1A). Co-administration of different adjuvants or TLR agonist, shifted tolerance induction to immunity by modulation of CD4^+^ T-cell responses. Poly I:C induced a strong IL-12-independent Th1 response. By contrast, curdlan led to the priming and induction of Th17 cells, illustrating the importance of the combination of antigen and adjuvants used to evoke diverse T-cell differentiation pathways.

## 4. Glycan-modification of Antigen to Modulate Immune-Responses

DEC-205, Clec9A and DCIR-2 have been studied using antibodies to dimerize the receptor as their glycan specificity is unknown. In contrast to these CLR, most CLRs are known to interact with glycans that are exposed on pathogens or on self glycoproteins. Therefore, glycan-structures can also be used as targeting motif to bring antigen to specific CLRs on DCs. The use of glycans for *in vivo* DC-targeting purposes has advantages over CLR-specific antibodies as glycans, many glycans are non-immunogenic and of self-origin, or can be produced synthetically in large scale using simple or complex chemistry depending on the complexity of the glycan structure [58]. Alternatively also glycan structures that are not of self-origin can be used that mimick for instance pathogenic glycan structures, that may also target CLRs with high affinity but are not of self-origin and may have immunogenic effects. In contrast, the production of humanized antibodies is expensive and can be immunogenic in patients. Furthermore, antibodies themselves are glycosylated, which is dependent on the cell line used for antibody production, and can induce unfavourable immune responses [59]. A possible drawback of using glycans could be that glycans by themselves have lower binding affinity to CLRs compared to antibodies. However, the glycan of choice can be designed to a scaffold as such that it is presented in a multivalent fashion to CLRs, mimicking the glycan composition on pathogens, leading to a higher binding affinity [60]. The potential of glycan-modification of antigens leading to immune modulation has been shown using different CLR like: MR, DC-SIGN and MGL.

The use of natural ligands to target the MR has been successful. The targeted delivery of mannose-or mannan-containing antigens to MR in combination with TLR stimuli leads to enhanced MHC class I and class II presentation as reviewed in Keler [61]. Also MR-targeting using sulphated glycans and GlcNAc-modified antigen resulted in a further increase of antigen presentation to CD4^+^ and CD8^+^ T-cells in a MR-dependent manner [62]. Furthermore, injection of DCs, *ex vivo* targeted with conjugates of glycan modified tumor antigen MUC1 (oxidized mannan-MUC1), in mice lead to in the generation of high frequencies of MUC1-specific CTL and protection from tumor challenge [63,64]. These studies formed the basis of clinical trails using oxidized mannan–tumor antigen conjugates to target MR. A pilot phase III study with stage II early breast cancer patients receiving oxidised mannan-MUC1 resulted in a significant drop in cancer recurrence: 0% of treated patients had tumor recurrences compared to 27% in placebo-receiving patients. Most of them had measurable antibody-responses against the MUC-1 and some of them had T-cell responses [65].

Another CLR investigated for DC-targeting using glycans is DC-SIGN. The carbohydrate specificity of human DC-SIGN has been well documented: DC-SIGN has affinity for both high-mannose- and fucose-containing glycans. These glycans can be exposed on certain self-antigens or pathogens and recognition by DC-SIGN results in antigen internalisation and subsequent processing, as well as signalling [31,66,67]. A recent study showed the capacity of glycan modified antigens to target DC-SIGN on DC using the human DC-SIGN transgenic mice [68]. Modification of antigen with DC-SIGN-specific carbohydrate structures induced strong induction of MHC class II mediated presentation to antigen specific CD4^+^ T-cells and MHC class I mediated cross-presentation to CD8^+^ T-cells [68]. Moreover, targeting of human DC-SIGN with specific antibodies also elicited strong CD4^+^ and CD8^+^ T-cells responses *in vitro* and in a humanised mouse model [14,69,70]. It would be very interesting to directly compare the potency of antibody-targeting to the targeting of CLR using glycans.

The CLR MGL expressed on DCs and macrophages has also recently been investigated. In humans, MGL interacts with terminal GalNac epitopes, e.g., tumour associated Tn antigens, and can efficiently internalise antigen for presentation to CD4^+^ T-cells [71]. In mice, 2 homologues of MGL are expressed, mMGL1 and mMGL2, recognizing Lewis^A^ and Lewis^X^ or GalNAc-structures respectively [38]. Antigen targeting to mMGL1 and mMGL2 using antigens modified with Lewis^X^- and GalNAc-structure respectively, resulted in enhanced cross-presentation to antigen-specific CD8^+^ T-cells [72,73], and unpublished observations). MGL1-targeting did also result in an increase of antigen-specific IFNγ-producing CD8^+^ T-cells *in vivo*; the *in vivo* effects of MGL2-targeting remains to be elucidated. Moreover, both MGL1 and MGL2 induced skewing of naïve CD4 T-cells towards T-helper 1 cells. However, there is a subtle difference: internalization of antigen via MGL2 induced enhanced CD4^+^ T-cell proliferation whereas MGL1-mediated internalization did not. If this has consequences *in vivo* remains to be investigated. Also other studies confirmed the induction of CD4^+^ T-cells upon MGL2-targeting using GalNAc [74,75]; however, no cross-presentation and CD8^+^ T-cell proliferation was observed. This could be due to differences in the nature of antigen used, resulting in differential intracellular routing. Furthermore, our studies on MGL2-targeting using GalNAc-structures also demonstrate the importance of multivalency on immune-modulation. Murine MGL2 interacts with tumor-associated antigen MUC1 similar to human MGL. It has been reported that the uptake of heavily glycosylated tumor-antigen MUC1 blocks intracellular processing and presentation and thus the induction of CTL responses [76,77]. In contrast, in our study where we conjugated only 1–2 moieties of GalNAc to each ovalbumin (OVA)-molecule, opposite effects were observed, that lead to a beneficial enhanced antigen uptake and presentation. Alternatively, a study on MR-receptor targeting showed that multi-branched mannosylated ligands were more efficient in targeting DCs compared to monomannosylated antigens [60], indicating that multivalency of glycan-CLR interaction is beneficial for antigen uptake and processing. These data imply that fine-tuning of the glycan-density on antigen is important, to find the balance between inducing the most potent immune-response required without tilting towards the induction of tolerance (Figure 1B).

## 5. Improvements

Many studies, using both antibody-antigen and glycan-antigen conjugates, have shown that the modulation of immune responses is possible via targeting of antigen to CLR. However, early clinical trials using the transfer of tumor antigen-loaded DCs have shown that in most patients immune-responses were detectable; However, clinical response were often lacking [78]. Advancing knowledge on the molecular mechanism of cross-presentation and understanding which DC subset is prone to cross-present antigens, as well as improving the formulation of the vaccine avoiding tolerance induction will lead to better *in vivo* efficacy of DC-based therapies. In the next section we will discuss improvements that can be made to ensure tumor eradication when using DC-based vaccination.

## 6. Skewing Antigen towards Cross-Presentation

The presentation of exogenous antigens in MHC-class I to CD8^+^ T-cells, a mechanism called cross-presentation, is crucial in anti-tumor vaccine development. Although this mechanism is under extensive research, it is currently still under debate. Various models for cross-presentation of antigens have been contemplated. There are two main pathways: the “cytosolic pathway” and “vacuolar pathway”, each with their own routing of antigen and required molecular interactions [79,80].

The mechanism by which antigen is internalized is important as it influences whether and via which pathway an antigen is cross-presented. Ovalbumin (OVA) which enters the DC via MR-mediated endocytosis is cross-presented, whereas OVA taken up through pinocytosis, enters the CD4^+^ T-cell route [81]. However, this MR-induced cross-presentation depends on the presence of endotoxin. When TLR-signalling molecules were knocked-out, cross-presentation was abolished. Furthermore, the physical form of the antigen seems to be of importance: OVA absorbed onto inert iron or polystyrene particles is cross-presented by the cytosolic pathway, whereas OVA encapsulated into PLGA-particles uses the vacuolar pathway [82]. It also has been suggested that the size of the antigen-particles is important [83,84]. Moreover, the nature of the receptor involved in endocytosis of antigen seems to be crucial for the routing of antigen intracellularly. In contrast to native OVA, which is taken up via the MR, glycan-modified OVA targeted to mMGL (both mMGL1 and mMGL2) resulted in TAP- and Cathepsin S-independent processing and very efficient cross-presentation of low amounts of OVA to CD8^+^ T-cells (unpublished observations).

Furthermore, antigen can also be artificially forced into the cross-presentation route by usage of saponin-based adjuvants. Saponin-based adjuvants break down phagosomes where antigen is located, translocating antigen to the cytosol, making it available for direct MHC class-I presentation [85,86].

Elucidating downstream pathways of antigen-routing and co-signalling of CLRs will result in knowledge about what intracellular signal decides cellular localization and if T-helper cells, CTLs or antibody responses is induced. This information is crucial for the design of better targeted therapies.

## 7. Superior APC for Targeting?

Preferentially, the antigen should be targeted to DCs only and more ideally to only those DC that is specialized to cross-present antigen. This will reduce the amount of antigen needed to elicit immune responses, which is crucial when the amount of antigen is limiting. Especially, it has been proposed that macrophage targeting should be avoided because of rapid degradation of antigen in lysosomes, which does not favour cross-presentation. Moreover, components allowing cross-presentation are expressed at low levels by macrophages.

However, there are also beneficial roles described for macrophages that cannot be ignored. Although macrophages are not ideally equipped for cross-presentation they are described to be able to cross-present antigen *in vivo* at least in some situations [87]. Furthermore, it has been described recently that a subset of CD169^+^ macrophages is present in the subcapsular sinus of the lymph nodes [88]. These macrophages are capable to take up and efficiently cross-present large antigens to T-cells, inducing an immunogenic response. Furthermore, it has been shown that metallophilic marginal zone macrophages (MMM) and CD8^+^ DCs collaborate in the spleen to induce CD8^+^ T-cell responses. Blood borne antigens are taken up by the MMM and may transfer antigen to splenic CD8^+^ DCs for cross-presentation and activation of cytotoxic T lymphocytes [89]. Also, peptide-loaded macrophages have been reported to show similar potency as DCs to stimulate naive CD8^+^ T-cells *in vivo*, which then develop into effectors and memory T-cells [90]. Taken together, because the receptor that solely targets DCs is yet to be discovered, and because macrophages are a substantial APC-subset present in the skin, it would be beneficial to study whether including macrophages in a DC-targeting vaccination strategy is better or worse for anti-tumor responses. It would be interesting to see whether cross-presentation skewing saponin-based adjuvants like ISCOMATRIX will also induce efficient cross-priming of CD8^+^ T-cells by macrophages [91].

Although macrophages can be beneficial in the induction of anti-tumor responses, the focus of targeting should lay on DCs, and preferably the DC subset that is most potent in achieving the most optimal anti-tumor response. DCs display delayed lysosomal acidification and proteolytic activity which favours cross-presentation. DCs are a heterogeneous population that consists of several DC-subsets which express different sets of pattern recognition receptors, have differential capacity to take up and present antigens and produce different cytokines upon TLR-stimulation [15,92,93]. This results in the induction of distinct types of immune responses. Targeting antigen to a receptor expressed on ‘the right DC-subset’ is required for the induction of the desired cross-presentation and induction of cellular responses. In mice, it has been shown, using antibody-targeting that not the targeting receptor but the targeted DC-subset is crucial for optimal CD4^+^ and CD8^+^ T-cell priming [13,44]. If this applies also to glycan-targeting remains to be established.

When intravenous injection is used, antigen travels through the bloodstream encountering DCs in the blood or spleen. In blood, three different DC-subsets are identified, namely: BDCA1^+^-, BDCA2^+^-, and BDCA3^+^ DCs. BDCA1^+^ DCs express a large array of TLRs (TLR 1,2,3,4,5,6,7,8, and 10) and upon stimulation with TLR-ligands produce pro-inflammatory cytokines [94,95]. BDCA3^+^ DCs are recently described to be the homologue of the mouse CD8^+^ DC which is specialized in cross-presentation. This DC-subset does express CLEC9A and TLR3 and -8 [56]. They potently cross-present antigens upon co-stimulation of TLR8 [15]. Although this sounds promising, if targeting of these cells *in vivo* will induce immune responses remains to be elucidated as these BDCA3^+^ DCs compel about 2% of the DC-population in blood. BDCA2^+^ pDC are specialized in anti-viral responses and upon viral (TLR 7-9) stimulation produce high amounts of type-I interferons [96]. Also pDCs are described to cross-present antigens [97,98]. Furthermore, they could add to the pro-inflammatory milieu needed for the induction of potent anti-tumor responses by the production of type-1 interferons. How all DC-subsets in blood contribute to modulating immune-responses is not yet known, and in view of *in vivo* CLR targeting it will be most efficient to target DCs in the skin.

The most common route of vaccination is subcutaneous injection in the skin where antigen will encounter local DCs. Here, different subsets can be targeted with different outcome of immune responses. Dermal DCs contain a large subset which is CD1a^+^ and a smaller CD14^+^ subset (about 10% of dermal DCs). CD14^+^ dDCs express a large array of CLRs like DC-SIGN, DEC-205, Dectin-1, DCIR, LOX-1 and Clec6. They also express TLR 2,4,5,6,8 and 10 [99]. The epidermis harbours Langerhans Cells (LCs), which only express the CLRs Langerin and DCIR and the TLRs 1,2,3,6, and 10 [100]. Recently, it has been shown that LCs can cross-present antigens to CD8^+^ T-cells *in vitro* as well as *in vivo* in mice [13]. Furthermore, a direct comparison of LCs and CD14^+^ dermal DC *in vitro* revealed that LCs are superior in inducing CD8^+^ responses whereas CD14^+^ dDCs were specialized in priming CD4^+^ T-cells into Follicular helper T-cells, inducing B-cell differentiation into antibody producing plasma cells [101]. However, if this division of labour is also operating *in vivo* still needs to be investigated.

## 8. Nanotargeting Systems

Some improvements can be made by optimizing the vaccine formulation, such as encapsulating antigen in a particulate and addition of a proper adjuvant. The use of nanotargeting systems has been shown to improve efficacy of targeting tremendously. Several delivery systems are being investigated under which metallic nanoparticles, polymer microparticles and liposomes. These systems resemble pathogens in size and can amplify immune responses. Furthermore, several nanotargeting systems protect antigen from non-specific degradation and form depots and aggregates of antigen, enhancing uptake by DCs. Using this technology both the antigen, the DC-targeting component and the adjuvant can be incorporated in one particle. Also, the particle used for DC-targeting will affect internalization mechanism and consequently the pathway of presentation. Ovalbumin absorbed onto inert particles is cross-presented via the cytosolic pathway, whereas ovalbumin encapsulated into microparticles uses the vacuolar pathway [82]. Also, liposomes can be designed to force antigen into either the MHC class I or11 presentation pathway [102]. The development of early endosomes into late endosomes/lysosomes coincides with a decreasing pH gradient, and liposomes were generated with different pH-sensitivity. This leads to breakdown of the liposome and release of antigen in either early endosomal or late endosomal/lysosomal compartment, facilitating either cross-presentation or MHC-class II presentation, respectively. Furthermore, the MHC class I and II presentation of antigen occurred through pathways having distinct molecular and proteolytic requirements [102].

Multiple studies have shown the contribution of nanotargeting systems in amplifying T-cell- as well as B-cell responses [103-105]. In these studies the uptake of the particles is at least partly due to pinocytosis or phagocytosis. These forms of endocytosis are not APC-specific and will lead to non-specific uptake of antigen by neighbouring cells, potentially leading to unwanted side-effects. In a phase I/II study which included stage II-IV melanoma patients receiving TAA-containing virus-like nanoparticles loaded with a TLR-9 ligand, it was shown that the vaccine was well tolerated [106]. These nanoparticles were not targeted to DC and taken up by phagocytosis. Although a-specific uptake seems to induce no significant side-effects, it is preferred to add a targeting component to the nano-targeting systems. In our own studies, we used glycan-coated liposomes to specifically target several CLRs, and avoided non-APC uptake. Furthermore, CLR-targeting using these glycan-coated liposomes is 60 times more efficient than targeting of a soluble glycan-antigen targeting to DC (unpublished observations).

## 9. Adjuvants

The development of an anti-tumor vaccine stands with the induction of the right pro-inflammatory response, avoiding the induction of tolerance. CLR-mediated uptake and presentation of antigen in general does not result in maturation of the DC (with exception of Dectin-1 and -2) and this lack of maturation will generally lead to tolerance. For the induction of a pro-inflammatory response, the DC must be fully activated and ‘licensed’. This is achieved by incorporation of a danger signal or so-called adjuvants, which guarantees the induction of a pro-inflammatory response. These adjuvants include TLR-agonists, inflammatory cytokines and agonists of co-stimulatory molecules. These adjuvants will not only modify the magnitude and type, but also improve the quality of the induced response. Most TLR-agonists can induce antibody responses, but have different capacity to induce CTL and T-helper responses. TLR-2 triggering leads to the induction of T-reg rather then Th-1 which is needed for anti-tumor immunity [107,108]. Furthermore, the activation of combinations of TLRs, like for example TLR4 with TLR7/TLR8, can induce synergistic production of cytokines inducing T-helper 1 responses [109]. Recently it was shown that the co-stimulation of TLR4 and TLR7/8 leads to the synergistic increase of the production of antigen-specific neutralizing antibodies compared to single TLR-stimulation [110]. This was due to early and increased programming of B-cell and CD4^+^ T-cell memory and was dependent on the direct triggering of both TLRs on B-cells and DCs, as well as on T-cell help. A very interesting observation was that the superior responses were induced when the antigen and TLR were delivered in separate nanoparticles compared to one nanoparticle that included both the antigen and TLR stimulus. However, whether this also applies for the induction of CD8^+^ T-cells remains to be determined. Moreover, the CLR used to target the antigen to, can often also signal, which can lead to either synergistic or inhibitory effects [16]. The addition of a TLR4-ligand to glycan-modified antigens targeting DC-SIGN or MR lead to synergistic effects on cross-presentation [62,68]. However, when TLR4 was triggered in combination with MGL1-targeting cross-presentation was greatly diminished (unpublished observations). These observations illustrate that for every DC-targeting receptor it is important to identify which adjuvant is best to combine to obtain the desired immune response.

As an alternative to TLR-triggering, the stimulation of co-stimulatory molecules using agonistic antibodies can be used as an adjuvant. CD40 triggering on DCs leads to maturation and licences DCs to prime CD8^+^ T-cells, and is used in multiple studies to fine-tune responses [11,45,111,112]

## 10. Concluding Remarks

CLR targeting on DCs via antibody- or glycan-antigen conjugates has been proven to be promising tools to develop new vaccination strategies. However, the strategy can be further optimized by choosing the most optimal targeting-receptor, appropriate adjuvants and integrating all needed vaccine-components in already established delivery systems such as liposomes and nanoparticles.

Whether *in vivo* DC-targeting on its own is potent enough to induce complete tumor-eradication in patients remains to be established. Eventually, combination therapies will probably be needed, where CLR-targeting for the induction of potent immune responses is combined with the breakdown of the immunosuppressive tumor-milieu. In particular, suppressive cytokines in the tumor-environment and immune-inhibitory signals can be blocked using antibodies or soluble receptors. Furthermore, the depletion of T-reg cells and myeloid suppressor cells will add to the break-down of t immunosuppressive milieu. Combining these signals will lead to an effective anti-tumor response a long-lived memory.

## Figures and Tables

**Table 1.** Expression, glycan specificity and function of CLRs expressed on APC such as macrophages, DC and Langerhans cells (LC), from group II, V and VI, used for DC-targeting applications.

**Lectin**	**Synonyms**	**Expression in human**	**Expression in mice**	**Carbohydrate specificity**	**Ligands**	**Function**	**Reference**
DEC205 Group VI	CD205; LY75; CLEC13B	BDCA1^+^ cDCs; monocytes; B-cells; low levels on NK cells; pDCs and T-cells	Cortica­l thymic epithelium; multiple subsets of DCs: CD11c^+^ CD8^+^ thymic medullary/splenic/lymp h node DCs; Dermal/interstitial DCs and LCs	Unknown	Dead cells; Y.pestis	Endocytic receptor	[20-22]
MR Group VI	CD206; MRC1; CLEC13D	Tissue macrophages and a subpopulation of DCs; lymphatic and hepatic epithelium, kidney mesangial cells;	Tissue macrophages and a subpopulation of DCs; lymphatic and hepatic epithelium, kidney mesangial cells;	mannose; Fucose; GlcNAc and sulphated glycans	C. albicans; M. tuberculosis; T.cruzi; and several other pathogens; (pro)collagen; serum hydrolases; tissue plasminogen activator; neutrophil derived myoloperoxidase; sialoadhesin;	Role in homeostasis; endocytic receptor	[23-30]
DC-SIGN Group II (murine homologue is not functional)	CD209; CLEC4L	DCs; specific macrophage subsets		High mannose; Lewis-antigens; GlcNAc (on LPS)	HIV; M.tubercolosis; C. Albicans; S.mansoni; wide panel of other pathogens. ICAM3; ICAM2; CD66a; MAC-1	Endocytic and receptor; production of numerous cytokines and chemokines	[31-34]
huMGL Group II	CD301; CLEC10A; CLECSF14	Immature DCs; macrophages		Terminal GalNAc-structures	CD45; S.mansoni; filoviruses; adenocarcinomas	Endocytic receptor; regulation of effector T-cells	[35]
mMGL1 Group II	CLEC10A; CD301a		Macrophages; interstitial DCs; pDCs	Lewis X; Lewis A structures		Uptake receptor, role in apoptosis	[35-38]
mMGL2 Group II	CD301b		Macrophages, DCs in dermis, small intestines and lymph nodes	Galactose; GalNAc	tumor-associated MUC1	Endocytic receptor	[35-38]
Langerin Groups II	CD207; CLEC4K	LCs	LCs; CD103^+^ DCs and CD8α^+^ DCs;	Mannose; GlcNAc; fucose; galactose-6-sulfated glycans	HIV Fibroblast-derived type I procollagen	Uptake receptor; induction of Birbeck granules	[39-42]
DCIR Group V	LLIR; CLEC4A; DDB27; CLEC SF6	DCs; monocytes B-cells; neutrophils; granulocytes; dermal DCs; pDCs	CD8α^-^ splenic DCs	Unknown	HIV	Uptake receptor	[43-46]
CLEC9A Group V	DNGR-1	BDCA3^+^ DCs	CD8α^+^ splenic DCs; pDCs	Unknown	Dead cell-associated antigens	Uptake receptor; cross-presentation dead cell-associated antigens	[12,15,17]
Dectin-1 Group V	CLEC7A; CLECSF12; Beta-glucan receptor	LCs; DCs; pDCs; macrophages; B-cells; granulocutes; T-cell subset	Monocytes; macrophages; DCs; neutrophils	Beta-glucans	C.albicans; A. fumigatus; P. carinii; C. posadasii; M. audounii, T. rubrum; T-cells	Uptake receptor; induction of DC-maturation; respiratory burst; production of numerous cytokines and chemokines	[47-51]
